# Impact of parental chromosomal polymorphisms on the incidence of congenital anomalies and perinatal complications in a cohort of newborns conceived after ICSI + PGT-A

**DOI:** 10.1186/s12958-022-01012-2

**Published:** 2022-09-27

**Authors:** Freddy Rodriguez, Maria Cruz, Antonio Requena

**Affiliations:** 1 Rey Juan Carlos University, Calle Quintana, 2 - 3ª planta, 28008 Madrid, Spain; 2 Valencian Infertility Institute, IVIRMA Global, Madrid, Spain; 3 Instituto de Investigación Sanitaria La Fe (IIS La Fe), IVI Foundation, Valencia, Spain

**Keywords:** Chromosomal polymorphisms, Congenital anomalies, Perinatal complications, Preimplantation genetic testing, ICSI

## Abstract

**Background:**

To assess the association between chromosomal polymorphisms (CPM) with congenital anomalies and perinatal complications in a cohort of newborns from couples undergoing intracytoplasmic sperm injection (ICSI), trophectoderm biopsy, and preimplantation genetic testing for aneuploidy (PGT-A).

**Methods:**

A retrospective cohort of singletons conceived after ICSI, trophectoderm biopsy, and PGT-A cycles performed at IVIRMA clinics in Spain over 4 years was involved in the study. Newborns were classified according to the parental karyotype analysis: Group I: non-carriers, Group II: CPM carriers. Couples with chromosomal anomalies and instances when both partners were CPM carriers were excluded from the study. The groups were compared for several perinatal complications.

**Results:**

There was a significant decrease in the number of NB with complications in the carrier group compared to the non-carriers (19.7% vs 31.9%, *p* = 0.0406). There were no statistical differences among the two groups regarding congenital anomalies, preterm birth, alterations in birth length and weight, cranial perimeter, Apgar test score, or sex ratio (*p* > 0.05).

**Conclusions:**

Chromosomal polymorphisms appear to have no adverse effects on congenital anomalies or perinatal complications on newborns from ICSI + PGT-A cycles.

## Background

Infertility is a significant condition worldwide, affecting up to 10–15% of all couples of reproductive ages. In 2018, the International Committee for Monitoring Assisted Reproductive Technologies – ICMART – reported that an estimated eight million infants were born worldwide from Assisted Reproductive Technologies (ART) in the last 40 years [1]. The rapid growth in the use of these technologies, and the novelty of their methods, have raised concerns about the effects they could have on women´s health, and the health of their offspring.

Some studies performed on national registries in the United States, Australia, Denmark, Finland, Norway, and Sweden have found a higher incidence of congenital defects in ART infants compared to those conceived spontaneously [2–4]. The same conclusion was reached in different metanalysis [5, 6]. The causes of this increased risk are still unknown, though it has been linked to different factors, like the high levels of gonadotropins use on ovarian stimulation, the embryo culture process, and the cause of infertility itself [5].

It is well known that genetic anomalies are an important cause of human infertility [7–9]. Cytogenetic analysis can be used to investigate whether underlying chromosomal anomalies may account for bad obstetric outcomes and failure to achieve pregnancy. However, most infertility cases fail to reveal gross abnormalities and rearrangements in karyotype [10]. In addition to structural abnormalities, there are also polymorphic variants on chromosomes, considered by cytogeneticists as normal and harmless variants. They are known as heteromorphisms or chromosomal polymorphisms (CPM), and they occur in the general population without apparent functional or phenotypic impact on carriers [11, 12].

In recent years, several groups have investigated the prevalence of CPM in different ethnic infertile populations. Most of these studies have reported that the incidence is three to five times higher in the infertile population compared to the fertile population, indicating that CPM may have an impact on infertility [11–13]. CPMs have also been reported to be associated with recurrent spontaneous miscarriages, poor obstetric history, and idiopathic infertility [13–15]. Their prevalence among different genders has been reported by many groups. Among infertile couples, some researchers have found that men have a higher incidence of CPM than women. This suggests that these variants may play an important role in spermatogenesis [16, 17].

The effects of CPM on ART are contradictory. Some studies found no effect of these variants on ART outcomes [13], whereas others concluded that heteromorphisms were associated with adverse results [14, 16–18]. A decrease in the number of euploid embryos in infertile couples and egg donors carrying CPM compared to non-carriers has been previously reported [12, 19]. However, others groups studying the embryonic aneuploidy rate did not find any difference [20]. Furthermore, it has been reported that males carrying CPM are more likely to have multinucleated embryos compared to non-carrier males [21]. Regarding obstetric complications, it was reported that there is a slight increase in preterm pregnancies in CPM carrier women compared to non-carriers [15].

Given the possible effect that CPMs have on reproduction, the objective of this study was to determine the association between these variants and pregnancy outcomes after infertility treatment, especially the effect they have on congenital abnormalities and perinatal complications.

## Methods

### Study and control populations

A retrospective analysis was carried out on a cohort of newborns (NB) from infertile couples. All couples underwent ICSI + PGT-A treatments where the embryo transfer was performed between January 2015 and December 2018, at IVIRMA Global clinics located in Spain. They had one or two euploid embryos transferred and had a singleton pregnancy, with a live NB. All couples underwent karyotype screening before treatment. Exclusion criteria included couples with abnormal karyotypes, multiple pregnancies, gamete donation, cleavage stage biopsy, and females with an anatomical defect of the reproductive system. Data were anonymized and extracted from the digital platform for clinical information management of IVIRMA Global.

NBs were grouped according to parental karyotype analysis: Group I (control group) included 1,253 NBs with normal chromosome parents; Group II included 66 NBs with CPM carrier parents (37 NBs from female carriers and 29 from male carriers). NBs from couples with polymorphic variants of chromosomes in both males and females were excluded from the study. This study was approved by the Institutional Review Board at IVIRMA Global and by the Spanish Clinical Research Ethics Committee.

### Karyotype analysis

Chromosome analysis was centralized in a reference cytogenetic laboratory (Biokilab, Spain), performed by culture of peripheral blood lymphocytes stimulated with phytohemagglutinin and subsequent staining with trypsin-Giemsa (GTG bands). A total of 15 metaphases were evaluated for each of the subjects and the banding resolution was 400–550 bands per haploid set. Polymorphisms were included only when their size was greater or smaller than at least twice the size of the corresponding region on the homolog chromosome [18]. Heteromorphisms were reported according to the International System for Chromosome Nomenclature [22].

### Classification of chromosomal polymorphisms

An increase or decrease in the length of the heterochromatin on the long arm of chromosomes 1/9/16/Y were designated as qh + or qh-. An increase or decrease in lengths of the stalks on the short arm of the acrocentric chromosomes (D/G groups) was recorded as 13/14/15/21/22 pstk ± . Double and increased satellites on the short arm on the same chromosomal group could also be observed and were designated as pss and ps + . An increase in lengths of heterochromatin region on the centromere was recorded as cenh + . The pericentric inversions of chromosome 9 were also considered chromosomal polymorphism. Multiple variations consisted of more than one variant.

### Ovarian stimulation, embryo culture, and biopsy

Patients underwent ovarian stimulation using standardized protocols based on age, antral follicle count (AFC), antimullerian hormone (AMH), and body mass index (BMI). When 3 or more follicles reached 18 mm in diameter, ovulation trigger was performed with r-hCG (Ovitrelle®, Merck) or GnRH analog (Decapeptyl®, Ipsen Pharma). Intracytoplasmic sperm injection was performed in all cases, and fertilization was assessed 16–18 h after microinjection. Embryos were cultured using a sequential culture system (Global for fertilization, LifeGlobal) [23].

On day 3, all embryos were subjected to laser-assisted hatching, and on days 5–6, all non-arrested embryos underwent laser-assisted trophectoderm biopsy. All blastocysts were individually vitrified within 1–2 h after biopsy using Kitazato vitrification kit (Kitazato Biopharma) until PGT + A results were available. Chromosomal analysis was centralized in a reference genetic laboratory (Igenomix), using Array Comparative Genomic Hybridization (aCGH) or Next-generation sequencing (NGS) technologies [23, 24].

After testing, euploid embryos were thawed using a commercially available warming solution (Kitazato Biopharma) following the manufacturer’s instructions and then cultured for 2–6 h. Thawing of the blastocysts was prioritized based on the best quality before biopsy, and only blastocysts that re-expanded after warming were considered suitable for transfer.

### Embryo transfer and follow-up

No more than two embryos were transferred in all cycles, with single embryo transfer always recommended. Approximately two weeks after embryo transfer, blood levels of hCG were tested. Clinical pregnancy was then confirmed via ultrasound with the presence of a gestational sac and a fetal heartbeat at 6 weeks after embryo transfer. Obstetric control was conducted outside of IVIRMA facilities.

According to the routine follow-up process at IVIRMA Global, all clinically pregnant patients were contacted after at least one month of the expected due date to confirm their pregnancy outcomes and complete the follow-up chart. This included perinatal complications, date of birth, birth weight, birth height, sex of the babies, neonatal diseases, cranial perimeter, and birth defects. Preterm birth (PB) was defined as birth before 37 weeks of gestation, very preterm birth (VPB) was defined as birth before 32 weeks of gestation, and post-term birth was defined as birth during or after 42 weeks of gestation. Low birth weight (LBW) was defined as < 2,500 g, very low birth weight (VLBW) was defined as < 1,500 g, and high birth weight (HBW) was defined as weight over 4,000 g.

Congenital malformations and perinatal complications presented by NBs were categorized following the 10th edition of the International Classification of Diseases (ICD-10), chapter XVII for congenital malformations, deformations, and chromosomal abnormalities, and chapter XVI for conditions originating in the perinatal period, which includes pathologies that have their origin before birth and up to the first 28 days after delivery, even if the morbidity occurs later.

### Statistical analysis

Continuous data were compared using Mann–Whitney U test, and the results are presented as the Median (interquartile range). Categorical data are presented as percentages, and Fisher’s exact test was used for comparisons. All statistical analyses were conducted using Statistical Package for GraphPad Prism version 9.1.2, and *p* < 0.05 was considered statistically significant. As this is a retrospective study, there was no actual sample size calculation. According to the patient selection criteria and the number of records included in the database, a description was made of the statistical power that was possible to achieve with the available data. Considering the selection criteria and the study period, we believed to have more than enough statistical power to find clinically relevant differences that could be statistically confirmed.

## Results

A total of 1,319 live singletons were included in this study, 66 of them from CPM carriers couples (37 from female and 29 from male carriers). The incidence of chromosomal polymorphism variations in infertile couples was 5.0% (2.2% for men and 2.8% for women). The distribution of chromosomes with CPM was different between genders (Table 1). Polymorphic variants from chromosomes 1, 9, and 16 represented 59.5% in infertile women and 37.9% in infertile men. Those variants from acrocentric chromosomes represented 32.4% of all CMP in women and 41.4% in men. Polymorphic variants of chromosome Y represented 13.8% in men. The most common chromosome affected by CMP was chromosome 9, both in males (24.1%) and females (43.2%), with the variant inv(9) responsible for 17.2% in men and 29.7% in women. Multiple variants were present in 6.9% of men and 8.1% of women.

**Table 1:** Frequency of chromosomal polymorphism variation

**Karyotype**		**No. of males with Heteromorphisms** **(*n* = 1,319)**	**Freq** **(%)**	**No. of females with Heteromorphisms** **(*n* = 1,319)**	**Freq** **(%)**
Total		29		37	
Chromosomes 1, 9, 16		11	37.9%	22	59.5%
	1q +	4	13.8%	1	2.7%
	9q +	2	6.9%	3	8.1%
	9q-	0		1	2.7%
	9cenh +	0		1	2.7%
	Inv(9)	5	17.2%	11	29.7%
	16q +	0		5	13.5%
Acrocentric chromosome (D/G group)		12	41.4%	12	32.4%
	13 ps + /pss	1	3.4%	1	2.7%
	14 ps + /pss	3	10.3%	4	10.8%
	14pstk +	1	3.4%	0	
	15 ps + /pss	2	6.9%	0	
	15cenh +	1	3.4%	0	
	21 ps + /pss	2	6.9%	1	2.7%
	22 ps + /pss	1	3.4%	6	16.2%
	22pstk +	1	3.4%	0	
Y chromosome variation		4	13.8%	0	
	Yq +	3	10.3%		
	Yqs +	1	3.4%		
Multiple variations		2	6.9%	3	8.1%
	9qh + ,21 ps +	1	3.4%	1	2.7%
	21 ps + ,22 ps +	1	3.4%	0	
	inv(9)(p12q12),21 ps +	0		1	2.7%
	14pstk + ,14pstk +	0		1	2.7%

The basal data of the two groups were compared, and statistical analysis showed no differences for maternal age, BMI, years of infertility, or female basal AMH, (*p* > 0.05; Table 2). There was a significant decrease in the AFC in the carrier group (*p* = 0.0416). Furthermore, we analyzed the male and female carriers separately. The AFC was significantly decreased in the female carriers (8.0 (6.5 – 12.0), *p* = 0,0138) but not in the partners of the male carriers (11.0 (6.5 – 18.3), *p* = 0,7484), compared to the non-carriers (data not shown). There were no significant differences between the days of stimulation, FSH or HMG dose, estradiol (E2), and progesterone (P4) levels on hCG day, the number of oocytes retrieved or the number of oocytes MII (*p* > 0.05).

**Table 2:** Basal characteristics of infertile couples

	**Group I** **(*n* = 1,253)**	**Group CPM** **(*n* = 66)**	**p**
Maternal age (years)	38.0 (36.0 – 40.0)	39.0 (34.8 – 41.0)	0.1730
BMI (Kg/m^2^)	22.1 (20.4 – 24.6)	22.0 (20.5—23.6)	0.4778
Years of infertility (years)	2.0 (1.5 – 3.0)	2.0 (2.0 – 3.4)	0.8822
AMH (ng/ml)	1.3 (0.5 – 2.7)	0.9 (0.3 – 2.3)	0.2307
AFC (n)	12.0 (8.0 – 17.0)	9.0 (6.5 – 15.0)	**0.0416**
Days of stimulation (days)	11.0 (10.0 – 12.0)	11.0 (10.0 – 11.0)	0.9245
FSH dosage (UI)	1,800 (1,350 – 2,250)	1,650 (1,500 – 2,250)	0.8096
HMG dosage (UI)	825 (675 – 1,500)	1,029 (750 – 1,500)	0.1385
E2 level on hCG day (pg/ml)	2,280 (1,540 – 3,001)	2,184 (1,304 – 2,791)	0.2781
P4 level in hCG day (ng/ml)	0.64 (0.35 – 0.95)	0.65 (0.32 – 1.3)	0.6665
Number of oocytes retrieved (n)	12.0 (8.0 – 17.0)	12.5 (8.0 – 20.25)	0.3518
Number of MII (n)	10.0 (7.0 – 14.0)	10.0 (7.0 – 15.25)	0.5522
Sperm concentration (× 10^6^/mL)	35.0 (13.0 – 63.0)	34.0 (6.0 – 65.5)	0.4038
Total number of sperm (n)			
Sperm progressive motility (%)	40.0 (26.0 – 53.0)	38.0 (17.3 – 50.5)	0.1620
Total number of progressive sperm (n)	33.7 (8.2 – 79.8)	27.4 (2.9 – 98.7)	0.3606

Sperm parameters are shown in Table 2. There was no significant difference between groups for sperm concentration, the total number of sperm, sperm of progressive motility, or the total number of progressive sperm (*p* > 0.05). When analyzing male and female carriers separately, male partners of female carriers had similar sperm parameters to the control group (*p* > 0.05), while men carriers had lower sperm quality, though the difference was not significant (*p* > 0.05) (data not shown).

IVF outcomes are shown in Table 3. For fertilization rate, there was a significant difference between groups (*p* = 0.0429). When analyzing male and female carries separately, female carriers had similar fertilization rate to the control group (75.0 (61.5 – 86.0) vs 78.0 (65.0 – 88.0), *p* = 0.3494), but for male carriers, fertilization rate was significant decrease (73.0 (53.0 – 81.0), *p* = 0.0417). Regarding the number of good quality blastocyst per cycle, blastocyst formation rate, abnormal blastocyst rate and proportion of SET/DET, there were no statistical differences between groups (*p* > 0.05).

**Table 3:** Comparison of the ICSI + PGT-A outcomes among the three groups

	**Group I** **(*n* = 1,253)**	**Group CPM** **(*n* = 66)**	**p**
Fertilization rate (%)	78.0 (65.0 – 88.0)	75.0 (57.0 – 86.0)	**0.0429**
Good quality blastocyst (n)	4.0 (2.0 – 6.0)	4.0 (2.0 – 5.0)	0.7450
Blastocyst formation rate (%)	57.1 (40.0 – 75.0)	57.1 (41.5 – 77.5)	0.9308
Abnormal blastocyst rate (%)	50.0 (25.0 – 67.0)	50.0 (26.5 – 67.0)	0.8710
SET	74.2	100.0	0.1229

Perinatal outcomes for each group are presented in Table 4. There was a significant decrease in the frequency of NB complications in the CPM carrier group compared to the non-carriers (19.7% vs 31.9%, *p* = 0.0406). Furthermore, when analyzing male and female carriers separately, female carriers had significantly lower NB complications than the control group (13.5%, *p* = 0.0184), but not male carriers (27.6%, *p* = 0.6915). There were no differences between groups in Apgar test score at minutes 1 and 5, length and weight at birth, cranial perimeter, frequency of C-section, sex ratio, frequency of birth weight alterations, or duration of pregnancy.

**Table 4:** Comparison of perinatal outcomes among the three groups

	**Group I** **(*n* = 1,253)**	**Group CPM** **(*n* = 66)**	**p**
Apgar 1	9.0 (9.0 – 9.0)	9.0 (9.0 – 9.0)	0.4334
Apgar 5	10.0 (10.0 – 10.0)	10.0 (10.0 – 10.0)	0.9580
Length (cm)	50.0 (49.0 – 51.5)	50.0 (49.0 – 51.0)	0.4561
Weight (kg)	3,320 (3,005 – 3,600)	3,150 (2,908 – 3,635)	0.1497
Cranial Perimeter (cm)	35.0 (34.0 – 36.0)	34.6 (34.0 – 35.88)	0.9166
Congenital anomalies (%)	1.9	1.5	> 0.9999
NB with Complications (%)	31.9	19.7	**0.0406**
C-section (%)	45.4	38.1	0.2990
Very Preterm birth (%)	0.7	0.0	> 0.9999
Preterm birth (%)	7.0	3.0	0.3125
Post-term (%)	0.9	1.5	0.4742
Very Low Weight (%)	0.5	0.0	> 0.9999
Low Weight (%)	4.3	4.0	> 0.9999
High Weight (%)	5.4	6.0	0.7480
Sex ratio (boys/girls)	0.96	1.13	0.6137

Regarding congenital anomalies, there were no statistical differences between the groups. Interestingly, there were no congenital anomalies for female carriers (0.0%, *p* > 0.9999), but male carriers presented the highest frequency (3.5%, *p* = 0.4387). s

## Discussion

Chromosomal polymorphisms are normal variations of karyotypes. Different studies have shown a 3-to-fivefold increase in the prevalence of these variants in infertile population [11–13]. There seems to be an association between CPM and decreased fertility, poor obstetric history, and spontaneous miscarriage [16]. Determining the role of these variants in reproductive outcomes and gametogenesis of infertile individuals has been the goal of several publications, with controversial findings between studies. As far as we know, this is the first study to investigate the impact of these variants in perinatal outcomes and congenital anomalies on the offspring of CPM carrier couples who use assisted reproduction technologies to achieve term pregnancy. All subjects were infertile couples undergoing ICSI + PGT-A treatment, resulting in one live NB after the transfer of one or two euploids embryos.

This study did not find any significant difference in the incidence of congenital malformation in NBs between CPM carrier couples and couples with normal karyotypes, although there is a certain upward trend in male carriers. Additionally, these variants showed to have a positive impact on the incidence of perinatal complications in NBs, particularly in female carriers.

The fact that we did not observe a negative trend between perinatal complications and congenital anomalies when CPMs are present does not necessarily conflict with previous reports stating that these variants affect gametogenesis, embryogenesis, and IVF outcomes. As our study included only pregnancies with a live NB, it is likely that if parental heteromorphisms were implicated in generating abnormal embryo or negatively affecting fetal development, these cases would have been eliminated either at the euploid embryo selection after PGT-A, at implantation stage, during spontaneous abortion, or even in termination of pregnancy due to malformation. Previous reports state that CPMs are associated with a higher miscarriage rate [13, 16] To our knowledge, there have been no publications to date investigating the prevalence of these variants in fetuses that fail to reach term pregnancy.

No statistical differences were found in the number of good quality blastocyst per couple, blastocyst formation rate, or abnormal blastocyst rate. Based on these results, we can only infer that if CPM increases abnormal embryo rate, those aneuploid embryos stopped their development during early pregnancy, were discarded based on PGT-A results or they stopped their development before reaching the blastocyst stage. Previous reports state that CPMs were associated with higher rates of chromosomal abnormalities among blastomeres at the cleavage stage [12, 19]. Also, couples that did not have any euploid blastocyst to transfer were not included in this study and this may have an impact on the abnormal blastocyst rate from CPM carriers.

Our data show that CPMs significantly affect fertilization rate on carriers, particularly male carriers. This finding is in line with that reported in previous studies. A meta-analysis showed that during IVF/ICSI, CPM male carriers had a significant negative effect on fertilization rate, but not female carriers [25].

In the case of sperm parameters, no significant statistical differences were shown between groups, although male carriers had slightly lower sperm quality. This is consistent with previous reports, where CPM had a detrimental impact on sperm quality, indicating an impact on spermatogenesis [11, 12, 26].

Regarding maternal basal characteristics and stimulation cycle, no significant differences were found between groups. Only the AFC was decreased on CPM female carriers. However, the number of oocytes retrieved, and the number of mature oocytes did not show any difference.

There are some limitations to this study. First, we only evaluated couples with live NBs. This approach could result in missing infants who died during pregnancy or the perinatal period, and those pregnancies terminated due to malformations. Second, patients with adverse outcomes might be less willing to answer post-treatment questions. Third, cytogenetic analysis was not performed in the NBs, which could show a direct impact of CPM on embryo development, gestational outcomes, and perinatal complications. Fourth, we studied cycles from ICSI + PGT-A, in which only euploid embryos were transferred, which leave out the effect of CPM on aneuploidy rate. Finally, as this is a descriptive retrospective study with limited sample size and low incidence of congenital anomalies in the study population, it resulted in a low statistical power analysis (2,8%), which means that we cannot rule out a possible impact of CPM on congenital anomalies on babies conceived after ICSI- + PGT-A. Based on these limitations, further studies in similar settings and with a higher number of individuals are needed to confirm our findings.

## Conclusions

In summary, parental chromosomal polymorphisms do not appear to have a significant negative effect on congenital malformation development and perinatal complications when euploid embryos are transferred in ICSI + PGT-A cycles. Future studies, however, should evaluate the effect these variants have on pregnancy outcomes after infertility treatment when PGT-A is not performed.

## Data Availability

The datasets used and/or analyzed during the current study are available from the corresponding author on reasonable request.

## Abbreviations

Acgh: Array Comparative Genomic Hybridization
AFC: Antral follicle count
AMH: Antimullerian hormone
ART: Assisted Reproductive Technologies
BMI: Body mass index
CPM: Chromosomal polymorphisms
E2: Estradiol
HBW: High birth weight
ICMART: International Committee for Monitoring Assisted Reproductive Technologies
ICSI: Intracytoplasmic sperm injection
LBW: Low birth weight
NB: Newborn
NGS: Next-generation sequencing
PB: Preterm birth
PGT-A: Preimplantation genetic testing for aneuploidy
P4: Progesterone
VLBW: Very low birth weight
VPB: Very preterm birth
